# What We Should Consider for Full Densification when Sintering

**DOI:** 10.3390/ma13163578

**Published:** 2020-08-13

**Authors:** Suk-Joong L. Kang

**Affiliations:** Department of Materials Science and Engineering, Korea Advanced Institute of Science and Technology (KAIST), Daejeon 34141, Korea; sjkang@kaist.ac.kr

**Keywords:** densification, entrapped gases, pore entrapment, grain growth, grain boundary, microstructural evolution, fast firing, two-step sintering, spark plasma sintering, flash sintering

## Abstract

To fully densify a powder compact, we should avoid two things: (i) entrapment of insoluble gases within pores and (ii) entrapment of isolated pores within grains. This paper describes general directions for promoting full densification in view of the above two points. Emphasis is placed on ways to potentially prevent pore entrapment in terms of grain growth control. Currently available techniques that can enhance densification while suppressing grain growth are briefly described, and their major mechanisms are discussed.

## 1. Introduction

**Figure 1:**
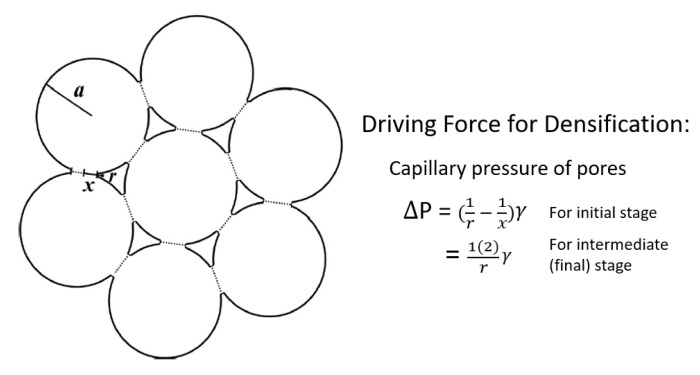
Idealized geometry of a powder compact to illustrate capillary pressure, ΔP, for densification in sintering models [2] (reprinted with permission from Wiley). *a*: radius of the particle; *x*: radius of the neck between two particles; *r*: radius of curvature of the neck (pore); and *γ*: surface energy.

When we sinter a powder compact, densification occurs under the driving force of the capillary pressure of pores [1]. For the same powder packing, the driving force increases linearly with a reduction in particle size, as illustrated in Figure 1 [2]. Reducing powder size can, therefore, be an approach for enhancing densification. However, this can also enhance grain growth and can change grain growth behavior. For a hypothesized system with no grain growth, the effect of powder size on densification is well documented as Herring’s scaling law [3]. In addition to particle size, size distribution is another parameter that affects densification, as it affects the packing of the powder, as well as pore size and distribution.

A number of investigations have been performed on the effects of particle size distribution on densification. Monosize powder was once thought to be beneficial for powder packing and densification. Liniger and Raj [4], however, demonstrated that monosize powder can cause enlargement of original pores and flaws because of locally enhanced densification in close-packed regions. Instead, they showed that a mixture of powders with two different sizes is beneficial for uniform densification. Several investigations have also reported that bi-modal and even multimodal distribution are beneficial for densification, while suppressing grain growth [5]. It seems that powder with a narrow size distribution or with a bi-modal distribution is beneficial for powder packing and densification.

The use of fine and size distribution-controlled powder, however, does not guarantee full densification of a powder compact. The causes of limited densification are (i) the presence of insoluble gases in isolated pores and (ii) the entrapment of isolated pores within grains. If insoluble gases are not present in the compact and pores are not entrapped within grains, full densification of any powder compact must be achievable.

In this paper, we discuss possible directions to avoid the above two causes of limited densification. In the discussion, we will cite only a few selected references to demonstrate the basis of the suggested directions.

## 2. Preventing the Entrapment of Insoluble Gases

**Figure 2:**
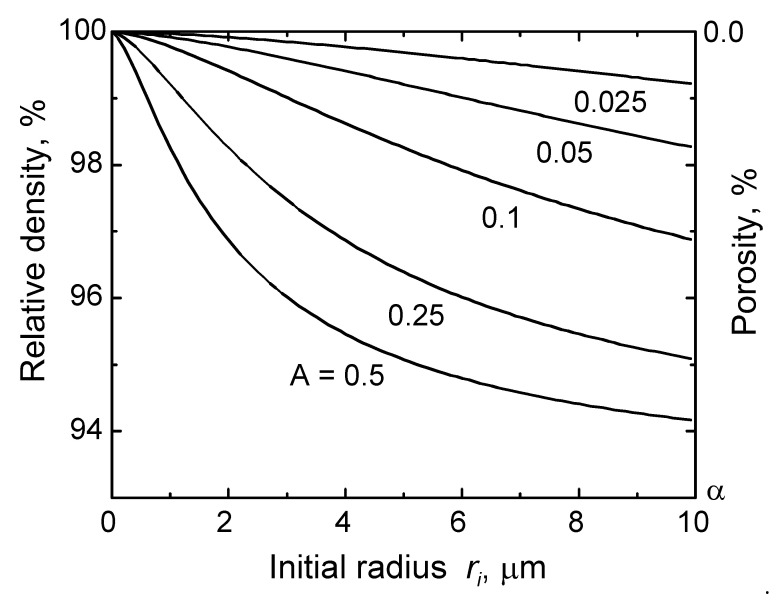
Maximum attainable density versus initial size of isolated pores, with initial porosity of α% [6] (reprinted with permission from Elsevier). The ordinate is scaled by assuming an initial density of 93% of the theoretical value. A = P_i_/2*γ* µm^−1^.

When insoluble gases are present and entrapped in a powder compact, gas pressure in the pores increases as densification proceeds. The effects of entrapped insoluble gases on the limit of densification can be estimated as functions of the initial size of the isolated pores, r_i_, and pressure, P_i_, of the gases entrapped in the pores at the moment of their isolation, as shown in Figure 2 [6]. The effects are reduced, and the achievable maximum density increases as the initial size of the pores is reduced, i.e., by reducing the size of the powder. During extended sintering, pore size can increase with pore coalescence, which is a result of grain growth. As pore size increases, the total volume of pores also increases due to reduction of the capillary pressure of pores; the volume of the compact increases, resulting in dedensification and even bloating of the compact [7].

Insoluble gases in a powder compact are either introduced from the sintering atmosphere itself or are generated during sintering, or both. There have been many investigations on the effects of sintering atmosphere on densification. A well-known example is that of Coble [8] during the development of translucent alumina (Lucalox^TM^). Sintering in hydrogen resulted in nearly full densification of the alumina, but sintering in air resulted in partial densification due to the entrapment of nitrogen, which is nearly insoluble in alumina.

When insoluble gases are generated during sintering, either by reactions between the powder and the sintering atmosphere, or by thermal decomposition of the powder itself, the insoluble gases are prone to becoming entrapped at the moment of pore isolation. Therefore, the sintering or presintering temperature, time period, and atmosphere must be chosen to avoid introducing/generating insoluble gases, or at least to eliminate the insoluble gases. An example can be found in the sintering of BaTiO_3_, where BaCO_3_ can be present as an impurity [9].

## 3. Pore Entrapment within Grains and Its Classical Analysis

The implication of pore entrapment within grains with respect to densification is well demonstrated in the classical investigation by Alexander and Balluffi [10]. They demonstrated that pore entrapment, which is the result of pore/boundary separation, was the limit of densification. Later, it was shown that entrapped pores were stable even under hot isostatic pressing of several thousand atm [11]. 

**Figure 3:**
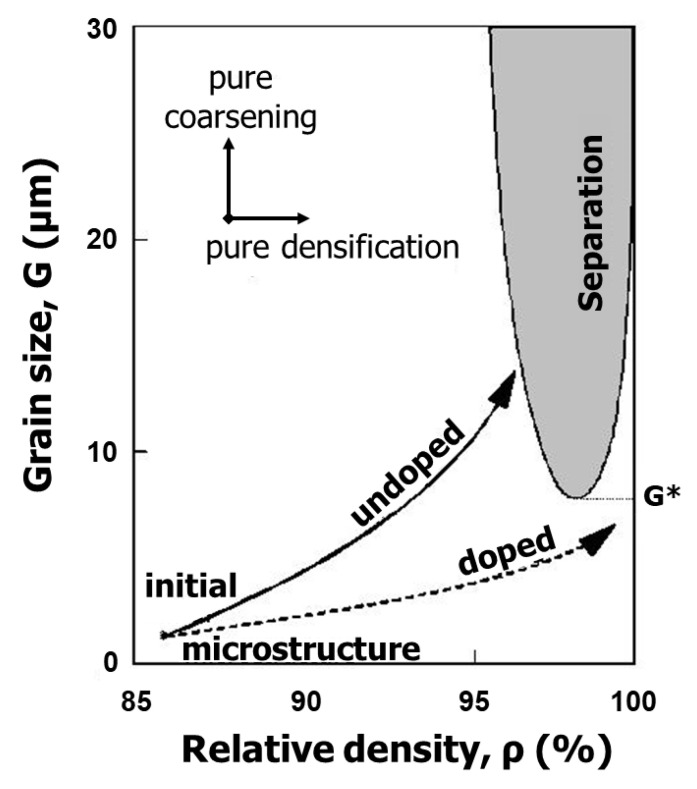
Schematic of the microstructure development map showing two different trajectories of undoped and doped samples [1,14] (reprinted with permission from Elsevier). It is assumed that the densification rate is raised by a factor of 10 by adding a dopant.

The phenomenon of pore/boundary separation (entrapment of pores) was studied theoretically from the 1960s to the 1980s for systems where the boundary migration for grain growth was linearly proportional to its driving force [12,13,14] (this is the condition for normal grain growth). Based on these analyses, a microstructure development map was introduced for different mechanisms of densification and grain growth. The analyses showed the presence of a pore/boundary separation region on a map of relative density versus grain size, such as the one shown in Figure 3 [1,14]. A general conclusion of the analyses is that pore/boundary separation (pore entrapment) can be avoided by enhancing densification while suppressing grain growth. In real systems, however, various types of grain growth, including abnormal grain growth, can take place. In such cases, the classical analyses should be no more valid for getting useful information about pore entrapment.

## 4. Suppression of Pore Entrapment: Grain Growth Control

**Figure 4:**
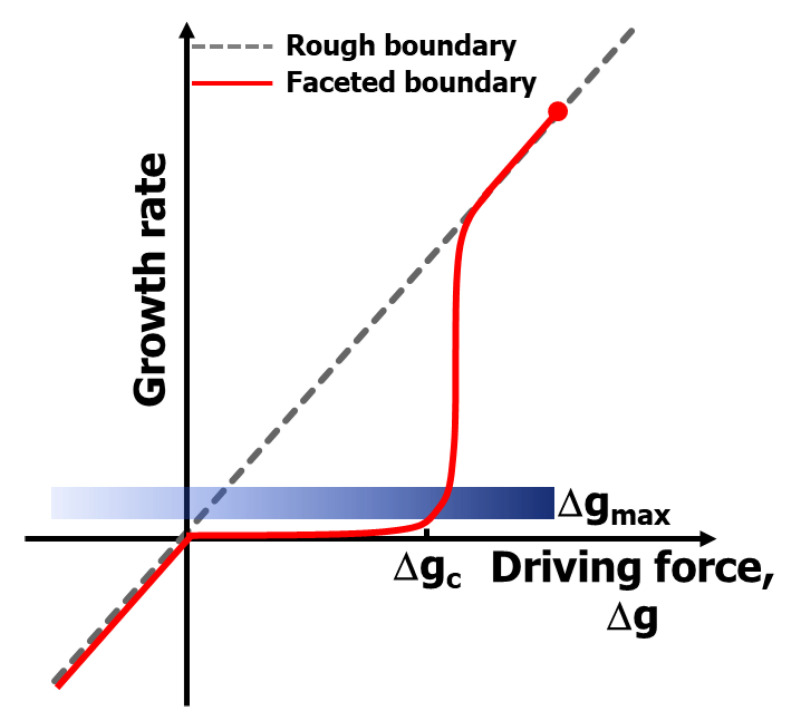
Schematic showing the mixed control model of boundary migration and grain growth [17] (reprinted with permission from Wiley). Δg_max_: maximum driving force for the largest grain; and Δg_c_: critical driving force for appreciable migration of a faceted boundary.

To avoid pore entrapment within grains, which is a result of grain growth, suppression/control of grain growth is essential. We found that grain growth behavior is largely governed by the morphology (atomic structure) of the grain boundary and can be controlled by changing the grain boundary structure, and powder size and distribution [15,16,17]. The basic idea of grain growth control relies on the new mechanism of boundary migration, namely the mixed control mechanism, which involves either jumping (diffusion) of atoms across the boundary or attachment (interface reaction) of the transported atoms on the surface of the growing grain [16,17,18]. Even for a partially faceted boundary, its migration is negligible if the migration driving force is smaller than a critical value (interface reaction control), while it is linearly proportional to a driving force larger than a critical value (diffusion control) (Figure 4) [16,17]. The critical value increases with an increase in faceting tendency, which is affected by temperature, atmosphere (e.g., oxygen partial pressure), and doping [15,19,20,21]. 

We were able to deduce the mixed-mechanism principle of microstructural evolution [16,17], which is based on coupling of the critical driving force, Δg_c_, for appreciable boundary migration, and the maximum driving force, Δg_max_, for the growth of the largest grain in the sample (Figure 4). The validity and generality of the microstructural evolution principle are supported by many investigations for various metallic as well as ceramic systems. The details of the principle and related experimental results can be found in the author’s previous publications [16,17,18,19,20,21].

## 5. Techniques for Enhancing Densification Relative to Grain Growth

There are several parameters that affect densification and grain growth, including external pressure, thermal cycle (temperature and heating rate), external field (such as an electric field), doping, and sintering atmosphere. The modification and application of these parameters has led to the development of unconventional sintering techniques.

### 5.1. Application of External Pressure

Probably the simplest way to enhance densification rate relative to grain growth rate is to apply external pressure. External pressure is additive to the capillary pressure of pores for densification [1,22,23]. External pressure can be applied uniaxially (hot pressing) or isostatically (hot isostatic pressing or gas pressure sintering). The benefit of applying external pressure for densification is well documented in the literature. Gas pressure sintering is known to be particularly useful for sintering materials with a high vapor pressure at their sintering temperature [24,25].

### 5.2. Modification of Thermal Cycle

**Figure 5:**
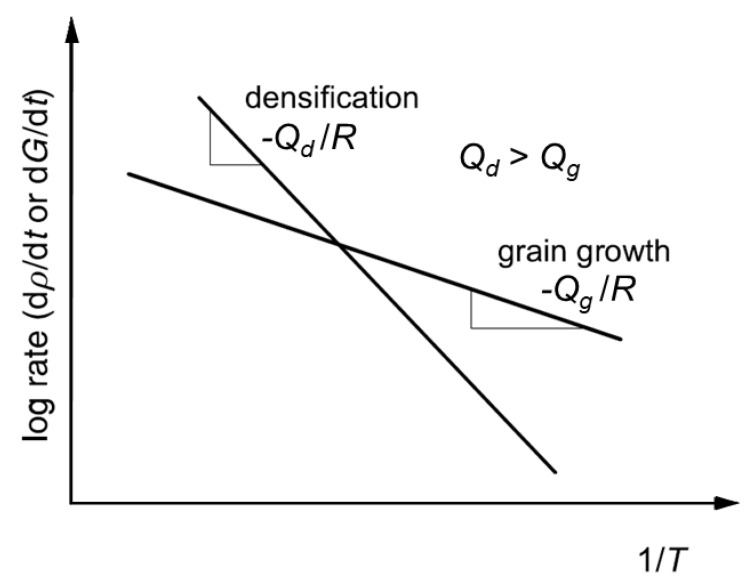
Temperature dependence of densification and grain growth rate for a material in which the activation energy of densification, Q_d_, is larger than that of grain growth, Q_g_ [1] (reprinted with permission from Elsevier).

Modifying the thermal cycle from the conventional one can considerably enhance the densification rate relative to grain growth rate. A typical example is the use of a much higher heating rate than the conventional one (called fast firing) [26,27]. In this technique, sintering usually proceeds at a temperature higher than the conventional sintering temperature for a short period of time. Fast firing seems to be a useful technique for fabricating many ceramic materials and components. The mechanism of enhanced densification with suppressed grain growth relies on the difference in activation energy between densification and grain growth [1,26]. For any system where the activation energy for densification is larger than that of grain growth, as shown in Figure 5 [1], fast firing can always be used to enhance densification and suppress grain growth.

**Figure 6:**
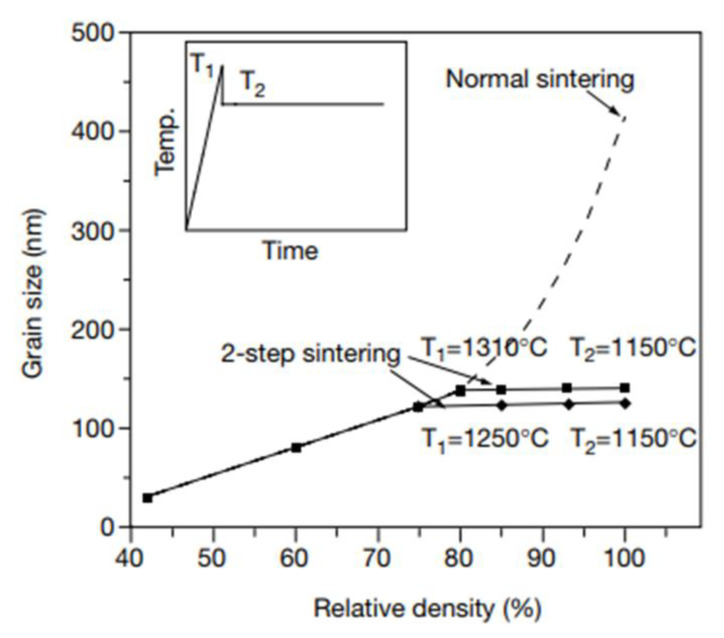
Microstructural evolution during two-step sintering of Y_2_O_3_ [28]. The inset shows a schematic of the thermal cycle in two-step sintering (reprinted with permission from Springer Nature).

Another technique that modifies the conventional thermal cycle is two-step sintering [28,29]. This technique consists of stepwise sintering at a high temperature for a short period of time, and then at a low temperature for a long period of time, typically the time period of conventional sintering. Grain growth can be much suppressed, as the example in Figure 6 shows [28]. Many investigations have reported the usefulness of two-step sintering, even for liquid-phase sintering [30,31]. The mechanism of two-step sintering seems, however, not yet clearly understood. For two-step liquid-phase sintering, an investigation reported that the mechanism was related to a reduction in the driving force for grain growth due to a change in the grain size distribution after a first-step sintering [31].

### 5.3. Application of an External Field

The effect of an external field has been widely studied. An example is microwave sintering. Many investigations have reported the beneficial effects of microwave sintering: fast heating and densification of the sample, and suppression of grain growth [32]. Several mechanisms for enhanced densification by microwave sintering have been suggested. The major mechanism in densification enhancement is fast Joule heating, which is the mechanism in fast firing. Non-thermal effects, such as ponderomotive force interaction and local anisothermicity, have been suggested as minor mechanisms [33]. Those secondary effects, however, do not seem to have been clarified.

Application of an electric field and current has been developed as a novel technique since the 1990s and has been intensively studied [34,35,36]. Spark plasma sintering (SPS), also called the field-assisted sintering technique (FAST), is a typical example. In SPS, high external pressure is applied together with an external electric field. Densification is greatly enhanced while grain growth is well suppressed. The mechanism in SPS has been studied intensively and is now accepted as being fast Joule heating, as in the case of microwave sintering. Secondary mechanisms may be active but are still unclear. It appears that SPS is a kind of hot pressing with a very fast heating rate.

Without any external pressure, a high electric field (current) alone can densify a sample in a very short period of time (a few seconds) in a technique called flash sintering [37]. It has been claimed that densification occurs far below the conventional sintering temperature. It is, however, difficult to measure the real temperature of a sample during flash sintering. Recently, Ji et al. [38] reported that the main mechanism of flash sintering is ultra-fast Joule heating. By using a self-propagating high-temperature synthesis (SHS) technique, the authors were able to heat up the sample very fast, as in the case of flash sintering, and achieved densification in a few seconds. Although there can be several mechanisms for flash sintering, its main mechanism seems to be ultra-fast Joule heating.

### 5.4. Addition of Dopants

In principle, the addition of dopants that create defects in slowly moving species with the fastest paths can enhance densification. There are, however, secondary and complex effects of doping, such as changes in grain boundary diffusivity/mobility, and grain boundary/surface energy and energy anisotropy. For many cases, the effect of dopants on densification does not follow the predictions of defect chemistry, most probably due to secondary and complex effects, which can drastically affect grain growth and behavior. 

Our investigations showed that the effect of doping, not only on densification but also on grain growth behavior, is closely related to a change in grain boundary morphology (structure) [19,20], similar to the effect of oxygen partial pressure [15,20,39]. The effects of dopant and oxygen partial pressure on grain growth could well be explained by the mixed control of boundary migration and the mixed mechanism principle of microstructural evolution.

## 6. Concluding Remarks

Full densification of a powder compact is achievable if we prevent (i) the entrapment of insoluble gases in the compact and (ii) the entrapment of pores within grains. For the former, the use of insoluble gas atmospheres must be avoided before pore isolation, and entrapping any generated insoluble gases must also be avoided during sintering. For the latter, grain growth must be suppressed/controlled. In particular, the fast growth of grains, typically abnormal grain growth, must be suppressed. Suppression/control of grain growth seems to be possible if we properly apply the mixed mechanism principle of microstructural evolution.

Several techniques are available for enhancing densification relative to grain growth. All things considered, fast heating of a compact seems to be a general direction for enhancing densification and suppressing grain growth. To achieve full densification, it would be desirable to use a proper technique that can enhance densification and to utilize the microstructural evolution principle for grain growth control.
